# Epilepsy seizure prediction with few-shot learning method

**DOI:** 10.1186/s40708-022-00170-8

**Published:** 2022-09-16

**Authors:** Jamal Nazari, Ali Motie Nasrabadi, Mohammad Bagher Menhaj, Somayeh Raiesdana

**Affiliations:** 1 Faculty of Electrical, Biomedical and Mechatronics Engineering, Qazvin Branch, Islamic Azad University, Qazvin, Iran; 2 Department of Biomedical Engineering, Faculty of Engineering, Shahed University, Tehran, Iran

**Keywords:** Seizure prediction, Epilepsy, EEG, CNN, Few-shot learning

## Abstract

Epileptic seizures prediction and timely alarms allow the patient to take effective and preventive actions. In this paper, a convolutional neural network (CNN) is proposed to diagnose the preictal period. Our goal is for those epileptic patients in whom seizures occur late and it is very challenging to record the preictal signal for them. In the previous works, generalized methods were inevitably used for this group of patients which were not very accurate. Our approach to solve this problem is to provide a few-shot learning method. This method, having the previous knowledge, is trained with only a small number of samples, learns new tasks and reduces the efforts to collect more data. Evaluation results for three patients from the CHB–MIT database, for a 10-min seizure prediction horizon (SPH) and a 20-min seizure occurrence period (SOP), averaged sensitivity of 95.70% and a false prediction rate (FPR) of 0.057/h and for the 5-min prediction horizon and the 25-min seizure occurrence period averaged sensitivity of 98.52% and a false prediction rate of (FPR) of 0.045/h. The proposed few-shot learning method, based on previous knowledge gained from the generalizable method, is regulated with a few new patient samples for the patient. Our results show that the accuracy obtained in this method is higher than the generalizable methods.

## Introduction

Epileptic seizures are transient signs or symptoms of abnormal, intense, and synchronous activities of the nervous system caused by electrical discharge from neurons. Approximately 50–60 million people worldwide have epilepsy [1]. In some patients, seizures may occur every few months or even every few years. Despite the low frequency of clinical symptoms, unpredictable seizures have profound effects on the patient's life [2].

Seizures, due to their unpredictability, often cause stress in the patient. More than 99.95% of the times, the patient is not having a seizure and should be able to live a relatively normal life, but the patient is always concerned that seizure can occur at any time and this affects their daily lives, often leading to anxiety and depression and lowering their quality of life [3]. The ability to accurately predict the seizures and to provide early warning before seizure occurrence can make significant changes in the lives of people with epilepsy, giving them greater confidence and freedom, as well as reducing sudden deaths in patients with epilepsy. In these cases, patients can take medication when necessary and not constantly. The electroencephalogram (EEG) signal has a higher temporal resolution than other brain imaging modalities and is used to predict more epileptic seizures. The EEG signal is a multivariate time series of a nonlinear and multidimensional system, so only complex nonlinear functions with a high degree of freedom can reveal the complex relationship between them. Today, there are significant advances in the field of machine learning, powerful algorithms such as the CNNs are utilized, since they have good results in natural language processing, object detection and classification and they acted very strongly to discover complex structures in data [4, 5]. One of the hypotheses for predicting epilepsy is that changes in brainwave patterns occur as we approach ictal. There are two perspectives for identifying these changes during the preictal interval that in one view, only the preictal interval is analyzed and it is compared to the threshold level [6]. In the second perspective, the distinctive patterns between the preictal and interictal intervals are identified and a binary classification is performed [7]. The commonality between these two perspectives is the extraction of the best features from the EEG signal. Many models have been used to predict seizures, yet the lack of efficient way for prediction still exists.

Most of the work done to diagnose and predict epileptic seizures is such that a number of time domain features such as median, mean, variance, standard deviation, maximum and minimum, etc.; frequency domain features such as power spectrum density, etc.; time–frequency domain features such as wavelet transform coefficients, Pseudo-Winger–Will, etc.; and chaotic features such as fractal dimension, approximate entropy and spectral entropy and correlation dimension, etc. are manually extracted. The combination of them is best described by the expert. In [6], they predicted epileptic seizures by introducing a similarity index based on symbolic dynamics techniques (statistical behavior of local extremes) with the sensitivity of 63.75% and FPR = 0.33/h for 21 patients from the Freiburg database and sensitivity of 96.66% and FPR = 0.33/h for eight patients.

In the last few years, various methods have been proposed to select the most appropriate combination of features and classifiers, including the results of extracting linear features from the EEG signal, such as autoregressive coefficients [8]. The emergence of dynamical systems theory introduced several nonlinear properties using features of Lempel–Ziv, noise level, correlation entropy complexity, and correlation dimension of intracranial EEG with the sensitivity of 86.7% and 92.9% with FPR = 0.126/h and 0.096 /h for SOP = 30 min and 10-s forecast horizon [9]. Spike rate used in this work [10]. For the CHB–MIT database, tailored feature extractions are customized and performed independently for each patient with a sensitivity of 98% and an FPR of less than 0.05/h [11]. With synchronization information, achieved a 95.4% sensitivity and FPR of 0.36/h [12].

A patient-specific method using the common spatial pattern (CSP) for feature extraction was reported with linear discriminant analysis (LDA) classifier with 89% sensitivity, FPR = 0.39/h, and SPH = 120 min for 24 CHB–MIT database patients [13]. In [14], the authors presented a patient-specific prediction algorithm using multiple features of spectral power of EEG signals and support vector machine (SVM) for classification, and reported sensitivity of 97.5% and FPR = 0.27/h for 18 Freiburg patients as well. In [15], authors have used the Recurrent Neural Network (RNN) to learn temporal dependencies between successive samples. Manual extraction of features is not only time-consuming but also imperfect. When faced with a wide range of data, it is challenging to engineer features and achieve high-level features. Generalized networks remove this constraint and allow data features to be extracted and learned without explicit structural information, and create an automated feature extraction path. In [16], they achieved FPR = 0.11–0.02/h and sensitivity 99% using the features of statistical moments, zero crossings, wavelet transform coefficients, PSD, graph theory, cross-correlation and using Long–Short-Term Memory (LSTM) for CHB–MIT data for 15–120 min SOP and zero SPH. In [17], using resting-state functional magnetic resonance imaging (rs-fMRI), EEG and LSTM achieved 96% sensitivity. In another study, they used convolution neural network on Functional near-infrared spectroscopy (fNIRS) and EEG data of 49 patients to reach 95.24–100% sensitivity [18]. In [19], using CNN, SVM for data set of 5 dogs and 2 patients, achieved 0.72% sensitivity. Used CNN and wavelet, was obtained sensitivity of 87.8% and an FPR of 0.147/h [20]. However, patient-specific feature-based tasks are generally high sensitivity and low FPR. Because the best feature combination is extracted and performed independently for each patient. However, the biggest problem with these methods is that they have to access a lot of preictal signals from each patient, which is difficult, especially in patients who have seizures late. The next problem with these methods is the manual extraction of features that is both time-consuming and error prone. In this work, to resolve these problems, we use the Few-shot learning method which requires a small amount of data from each patient and convolutional neural networks are also used to extract the feature, which eliminates the need for manual feature extraction.

The rest of the paper is organized as follows. We first describe the proposed method in the next section. Evaluation method, database and experimental results are given in Sect. 3. Section 4 presents discussion and comparisons and Sect. 5 finally concludes the paper.

## Proposed method

In this section, we will present the database, preprocessing, and the details of the proposed methods for predicting epileptic seizures.

### Database

EEG signals are recorded in two ways: intracranial EEG (iEEG) and scalp EEG. In the iEEG technique, the electrodes are located exactly on the brain and it is an invasive method, but in the scalp EEG method, the registration is done on the scalp and it is easy to register, so because it is non-invasive and more accessible, the scalp EEG is used for this work.

Boston Children’s Hospital (CHB)–MIT data set [21]. Included scalp EEG data from 23 subjects with 844 h of continuous EEG recording and 163 seizures. All signals were sampled at 256 samples per second with a 16-bit resolution. The EEG signals were captured with the use of 22 electrodes. In this study, we consider patients whose signal was recorded at least 30 min before the seizure. In this work we will use data from 18 patients and 16 common channels.

### Preprocessing

**Fig. 1:**
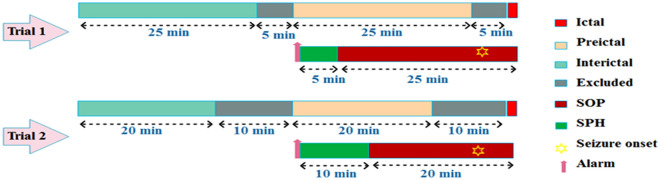
Preictal intervals used in Trial 1 and Trial 2

In this work, 16 raw EEG channels are used simultaneously. Two-dimensional (2D) CNN convolution is required. Our method is based on two-class classification to distinguish the preictal from the interictal. However, it is essential to know how long the preictal interval starts from the seizure onset and how long it lasts, because if the Seizure prediction horizon (SPH) is longer, the patient has enough time for coping and taking vital actions. The seizure occurrence period (SOP) should not be long so that the patient is less anxious. In trial 1, we consider the signal 5 min before the ictal to 30 min before the ictal, that is a 25 min interval that is considered as the preictal interval. This means SOP = 25 min and SPH = 5 min. In trial 2, we consider the signal 10 min before the ictal to 30 min before the ictal, that is a 20 min interval is considered as the preictal interval. This means that SOP = 20 min and SPH = 10 min as shown in Fig. 1. SPH is the space of between the alarm and the onset of the SOP. For the alarm to be true, seizures should begin after SPH and within the SOP. Passing the signal through a bandpass filter, we select the middle frequency of 0.5–100 Hz, and we eliminate a 60 Hz frequency power line noise via a notch filter.

After normalization, by passing a sliding window with a length of 5 s with variable overlap, segments with a length of 1280 samples are selected from the preictal and interictal range equally due to the balance of data in both classes. One of the essential issues in classification is the balance of the data set which means that each class has an equal number of data. Due to the fact that in the database used, the length of the preictal period is short in a number of patients. To select segments from preictal intervals with shorter lengths, the variable overlap is used in learning phase. It is noteworthy that overlapping segments are not used in the test. As a result, each segment is a two-dimensional matrix with a size of 16*1280, which means that each channel with a length of 5 s (1280 samples) is located in a row.

### Generalizable

The proposed generalizable method consists of a CNN architecture in first stage for feature extraction and a SVM for binary classification. Deep architecture allows the reuse of features (mid-level features that are shared between all classes). It has also the potential to create high-level features.

CNN is a feed-forward network inspired by the animal visual cortex. CNNs can detect complex structures in data. They require little preprocessing, meaning that the model itself is responsible for learning the features extracted manually in traditional algorithms [22]. Automatic feature extraction from raw data, without the need for prior knowledge and without the need for an expert or a specialist, as well as the concept of hierarchical learning in it, has made it essential to do this work. It has been shown that in the early layers, low-level features are learned, and as it deepens, higher level concepts are learned [20, 23]. In this model, Dropout and max-pooling layers are used to prevent overfitting [24]. The batch normalization technique is used in all layers for faster training and improved accuracy [25]. The model used has an end-to-end learning architecture, that is learning from the raw EEG without any feature extraction.

**Fig. 2:**
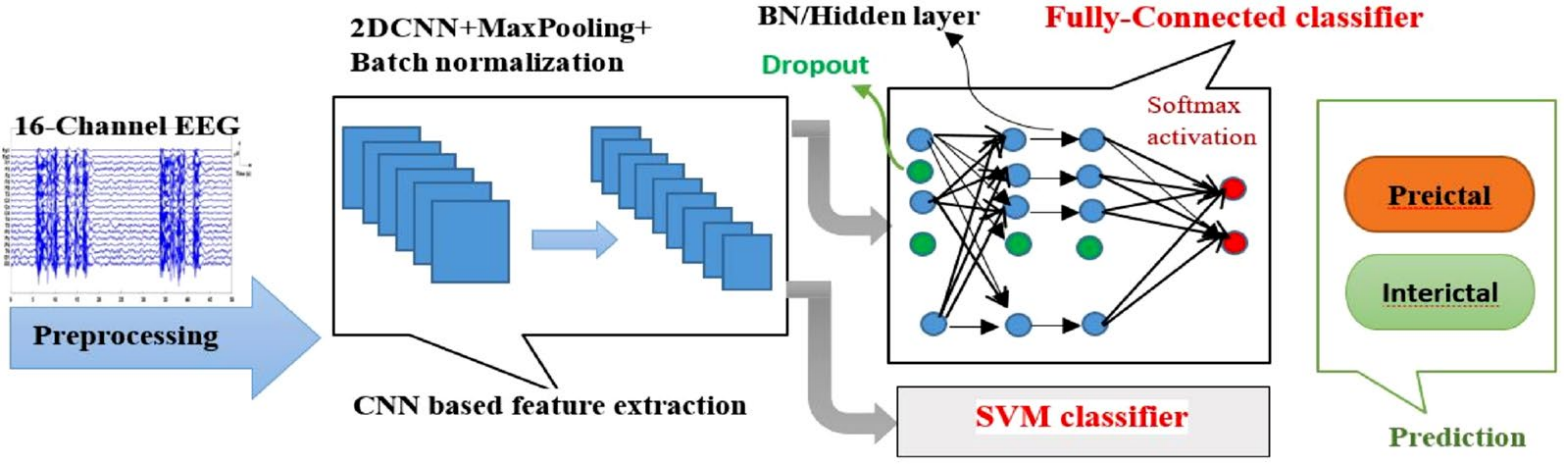
Proposed generalizable method

Two layers of the convolutional layer are applied to the input, which is an EEG signal with size (n * 16 * 1280), n is the number of input data sets. Each convolution layer contains a 3*3 kernel and single stride. Dropout and batch normalization is applied to all layers. A max-pooling layer of 2*2 size is then applied in with two strides. Afterward, the convolution output is first flattened and then an FC (fully connected) with two hidden layers and one output layer is used. In every two hidden layers, the Dropout and Batch normalization is performed. The two neurons of the last layers use the soft-max activation function and the rest of the layers use the ReLU activation function. In the second case, an SVM classifier is used instead of employing fully connected layers and a soft-max activation function. The generalizable method architecture is shown in Fig. 2.

### Few-shot learning

The main method in the discussion, which is the primary purpose of this work, is called few-shot learning (FSL), which we will examine.

Few-shot learning is a machine learning method that aims to achieve good performance with the help of limited data [26, 27]. FSL methods are trained with just a few examples of labeled data. FSL can quickly learn new tasks that have little data with the help of prior knowledge and reduce the effort to collect more data [28, 29]. FSL methods can be classified into three groups:

1. Data: Enhancing educational data sets using methods such as manual data augmentation in preprocessing or techniques such as Generative Adversarial Networks (GANs) to generate unrecognizable synthetic data.
2. Model: Reducing the number of network learnable parameters with methods, such as parameter sharing.
3. Algorithm: Using models that have already been trained for related tasks. Transferring prior knowledge from a pre-trained model to do the same work is known as transfer learning [30].

For the definition of transfer learning, denote the domain *Q* = {H, P(X)} for feature space H and marginal probability distribution P(X), where *X* = {× 1, …, xm} ∈ H. A task K included a label space Y and target predictive function *f*: H → Y, defined by *K* = {Y, f(x)} is learned by the training data consisting of pairs {xi, yi}, where xi ∈ X and yi ∈ Y. Given a source domain QS and learning task KS, a target domain QT and learning task KT, where QS ≠ QT or KS ≠ KT, transfer learning goals to modify the learning of the target predictive function fT(.) in QT using the knowledge in QS and KS [31].

**Fig. 3:**
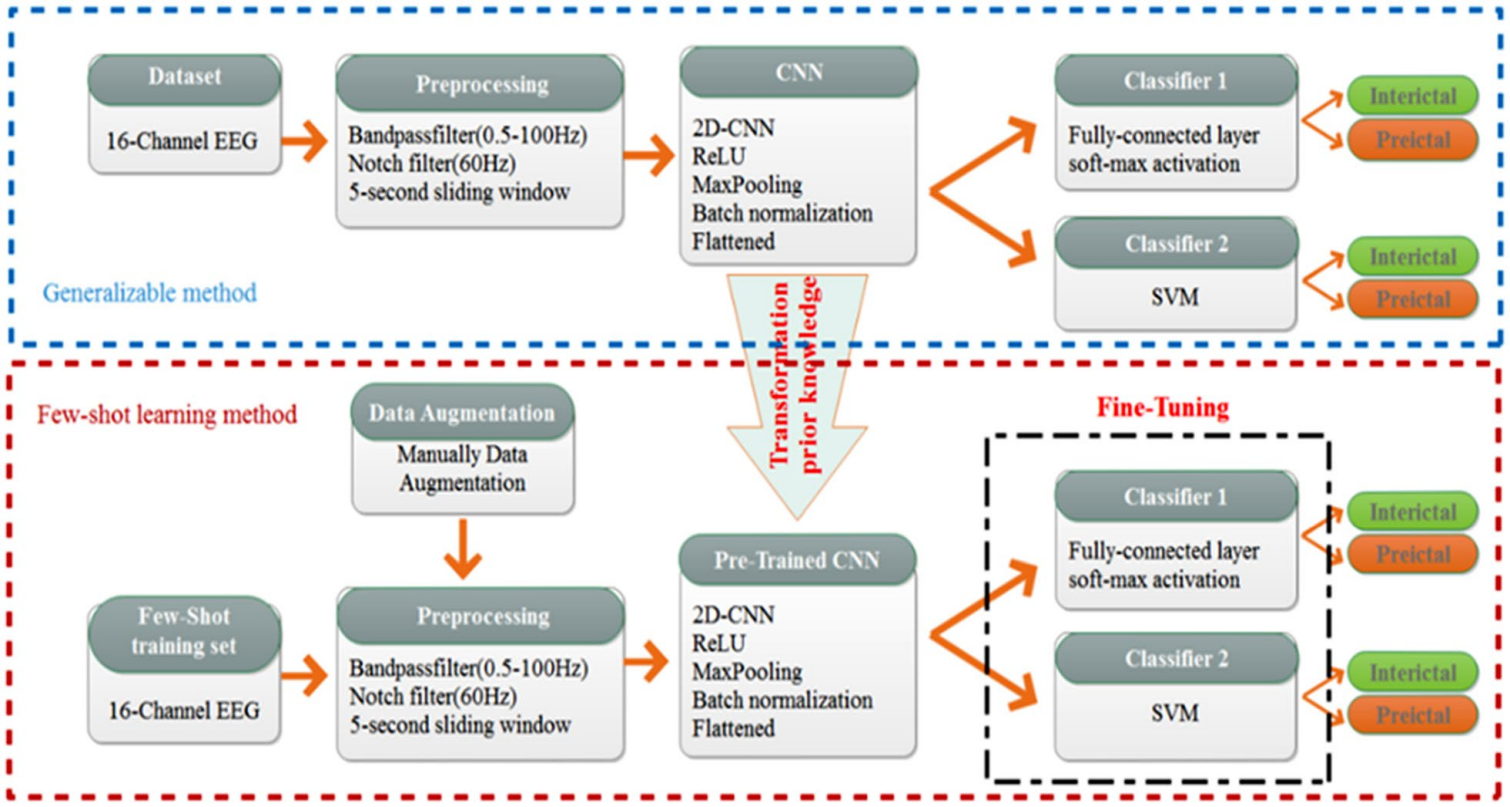
Proposed method block diagram

Transferring previous knowledge from the proposed generalizable method which has been trained with the data of 15 subjects in the CHB_MIT database is used to extract the feature. The weights of the convolution layers are not updated and the classifier layers are fine-tuned using only a few data from the patient. By freezing the weights of the CNN layers, we have reduced the number of learnable parameters and, in fact, implemented the concept of parameter sharing. A block diagram of both methods is given in Fig. 3. To increase the data manually, which was described in the pre-processing section, a slider window with variable overlap was used to increase the training data.

In this method, which is a patient-specific method, we use several methods for FSL. Using transfer learning methods, parameter sharing, Manually Data Augmentation together in this method has improved its performance.

The generalizable method includes the input of 15 patients who enter CNN after preprocessing and then use SVM or FC as a classifier. In the few-shot learning method, a new patient's data is entered to a CNN, which is the same as CNN used in the previous generalizable method, and it finally is applied to the classifier layers to update weights of the classifier layers for the new patient.

## Results and discussion

We define the following parameters to evaluate and compare these methods for different preictal intervals:

- Seizure prediction horizon (SPH) is defined as the interval between the alarm and the onset of the seizure occurrence period (SOP). For the alarm to be actual, seizures should begin after SPH and within the SOP.
- False prediction rate per hour (FPR/h) is the number of incorrect alarms per hour when they are positively predicted, but no seizure occurs in the SOP [7].
- Sensitivity is determined the percentage of rightly predicted seizures divided on the all seizures.
- Area Under the Curve (AUC) of the Receiver Operating Characteristics (ROC) curve It is one of the most essential evaluation metrics for checking any classification method’s performance. sections.

### Generalizable method results

Generalizable method that we trained with 15 patients from the CHB–MIT database. The evaluation results of this method with the leave-one-out cross-validation (LOOCV) method for trial 1 and trial 2 are shown in tables 1 and 2. The LOOCV technique uses all data except one for training and the residuum data for the method testing. This garlic is repeated N times; N presents the number of data folders. With this work all data will be used to train and test the method. The method error rate is equal to the average error rate per iteration. For comparison of the results, the average of sensitivity, FPR, and AUC for 15 patients are calculated and given in the table.

**Table 1:** Test results of the generalizable method for Trial 1 (SOP = 25 min, SPH = 5 min)

Patient name	Fully connected			SVM		
	Sensitivity (%)	FPR (/h)	AUC	Sensitivity (%)	FPR (/h)	AUC
Chb01	94.09 ± 0.2	0.013 ± 0.001	0.954 ± 0.004	98.78 ± 0.7	0.011 ± 0.006	0.984 ± 0.002
Chb02	50.00 ± 0.7	0.103 ± 0.006	0.901 ± 0.002	68.62 ± 0.1	0.100 ± 0.006	0.699 ± 0.004
Chb04	95.45 ± 1.2	0.110 ± 0.005	0.967 ± 0.005	90.72 ± 0.2	0.097 ± 0.006	0.973 ± 0.005
Chb05	71.36 ± 0.5	0.030 ± 0.009	0.786 ± 0.004	68.65 ± 0.4	0.121 ± 0.003	0.798 ± 0.003
Chb06	100.00 ± 0.3	0.051 ± 0.002	0.984 ± 0.012	86.92 ± 0.8	0.041 ± 0.006	0.894 ± 0.001
Chb07	81.54 ± 0.6	0.018 ± 0.005	0.912 ± 0.009	100.00 ± 0.0	0.023 ± 0.009	0.982 ± 0.007
Chb09	82.91 ± 1.1	0.032 ± 0.004	0.891 ± 0.021	98.32 ± 0.1	0.110 ± 0.007	0.996 ± 0.002
Chb14	62.98 ± 0.7	0.114 ± 0.006	0.919 ± 0.004	71.82 ± 0.6	0.050 ± 0.001	0.957 ± 0.008
Chb15	99.19 ± 0.7	0.023 ± 0.009	0.997 ± 0.007	90.02 ± 0.5	0.000 ± 0.006	0.949 ± 0.002
Chb17	81.73 ± 0.9	0.083 ± 0.001	0.924 ± 0.006	86.49 ± 0.2	0.097 ± 0.005	0.934 ± 0.004
Chb18	78.18 ± 1.4	0.112 ± 0.006	0.897 ± 0.001	71.22 ± 0.7	0.105 ± 0.002	0.798 ± 0.001
Chb19	99.09 ± 0.6	0.017 ± 0.005	0.998 ± 0.002	91.64 ± 0.1	0.001 ± 0.004	0.929 ± 0.005
Chb20	100.00 ± 0.8	0.040 ± 0.008	0.901 ± 0.007	89.84 ± 0.3	0.091 ± 0.001	0.897 ± 0.002
Chb21	77.72 ± 1.2	0.110 ± 0.008	0.867 ± 0.020	89.81 ± 0.5	0.106 ± 0.002	0.928 ± 0.003
Chb22	86.05 ± 0.1	0.135 ± 0.002	0.944 ± 0.001	98.10 ± 0.2	0.124 ± 0.006	0.967 ± 0.001
Avg	84.02 ± 0.7	0.066 ± 0.005	0.922 ± 0.007	86.73 ± 0.3	0.071 ± 0.004	0.912 ± 0.003

**Table 2:** Test results of the generalizable method for Trial 2 (SOP = 20 min, SPH = 10 min)

Patient name	Fully connected			SVM		
	Sensitivity (%)	FPR (/h)	AUC	Sensitivity (%)	FPR (/h)	AUC
Chb01	92.12 ± 0.3	0.024 ± 0.003	0.933 ± 0.005	95.84 ± 0.6	0.036 ± 0.005	0.969 ± 0.001
Chb02	83.05 ± 0.2	0.151 ± 0.002	0.891 ± 0.004	85.36 ± 0.2	0.120 ± 0.002	0.874 ± 0.005
Chb04	93.87 ± 0.4	0.090 ± 0.004	0.958 ± 0.005	91.25 ± 0.4	0.087 ± 0.005	0.984 ± 0.005
Chb05	75.54 ± 0.4	0.098 ± 0.002	0.892 ± 0.001	79.44 ± 0.5	0.134 ± 0.001	0.718 ± 0.002
Chb06	91.89 ± 0.7	0.097 ± 0.005	0.943 ± 0.015	100.00 ± 0.4	0.097 ± 0.005	0.997 ± 0.006
Chb07	83.35 ± 0.2	0.089 ± 0.001	0.907 ± 0.001	90.12 ± 0.1	0.114 ± 0.004	0.921 ± 0.004
Chb09	79.88 ± 0.6	0.087 ± 0.006	0.851 ± 0.007	88.52 ± 0.7	0.210 ± 0.003	0.864 ± 0.003
Chb14	95.18 ± 0.2	0.044 ± 0.005	0.912 ± 0.005	90.66 ± 0.6	0.077 ± 0.008	0.943 ± 0.004
Chb15	91.22 ± 0.1	0.073 ± 0.002	0.945 ± 0.006	91.65 ± 0.3	0.034 ± 0.001	0.951 ± 0.001
Chb17	67.83 ± 0.1	0.094 ± 0.001	0.902 ± 0.001	81.80 ± 0.1	0.058 ± 0.002	0.974 ± 0.005
Chb18	71.91 ± 0.5	0.124 ± 0.003	0.825 ± 0.003	75.41 ± 0.2	0.165 ± 0.005	0.795 ± 0.004
Chb19	94.02 ± 0.4	0.072 ± 0.004	0.976 ± 0.005	92.83 ± 0.1	0.084 ± 0.002	0.988 ± 0.002
Chb20	87.20 ± 0.2	0.079 ± 0.001	0.895 ± 0.002	88.25 ± 0.0	0.088 ± 0.003	0.926 ± 0.003
Chb21	70.98 ± 0.7	0.205 ± 0.002	0.798 ± 0.009	80.00 ± 0.4	0.211 ± 0.001	0.897 ± 0.005
Chb22	90.11 ± 0.2	0.121 ± 0.003	0.951 ± 0.002	96.23 ± 0.1	0.099 ± 0.002	0.943 ± 0.006
Avg	83.94 ± 0.3	0.087 ± 0.003	0.905 ± 0.004	88.49 ± 0.3	0.114 ± 0.003	0.916 ± 0.004

### Few-shot learning method results

To evaluate the few-shot learning method, used six seizures from subject Chb03 of which three seizures were used for fine-tuning the method and three seizures were excluded for method testing. We repeated the above experiment for subject Chb10 and subject Chb16 in the same way. The test results of few-shot learning method are given in tables 3 and 4.

**Table 3:** Test results of the few-shot learning method for Trial 1 (SOP = 25 min, SPH = 5 min)

Patient name	Fully connected			SVM		
	Sensitivity (%)	FPR (/h)	AUC	Sensitivity (%)	FPR (/h)	AUC
Chb03	96.18 ± 0.1	0.086 ± 0.006	0.963 ± 0.003	100.00 ± 0.5	0.056 ± 0.002	0.990 ± 0.003
Chb10	94.90 ± 0.4	0.061 ± 0.007	0.988 ± 0.001	95.76 ± 0.6	0.079 ± 0.001	0.984 ± 0.002
Chb16	98.54 ± 0.7	0.019 ± 0.006	0.984 ± 0.006	99.82 ± 0.4	0.000 ± 0.004	0.996 ± 0.005
Avg	96.54 ± 0.4	0.055 ± 0.006	0.978 ± 0.003	98.52 ± 0.5	0.045 ± 0.002	0.990 ± 0.003

**Table 4:** Test results of the few-shot learning method for Trial 2 (SOP = 20 min, SPH = 10 min)

Patient name	Fully connected			SVM		
	Sensitivity (%)	FPR (/h)	AUC	Sensitivity (%)	FPR (/h)	AUC
Chb03	94.18 ± 0.2	0.066 ± 0.001	0.944 ± 0.007	95.92 ± 0.1	0.070 ± 0.006	0.989 ± 0.003
Chb10	90.10 ± 0.8	0.069 ± 0.006	0.908 ± 0.002	93.42 ± 0.7	0.101 ± 0.005	0.975 ± 0.004
Chb16	92.24 ± 0.6	0.080 ± 0.002	0.937 ± 0.003	97.78 ± 0.5	0.002 ± 0.002	0.986 ± 0.002
Avg	92.17 ± 0.5	0.071 ± 0.003	0.929 ± 0.004	95.70 ± 0.4	0.057 ± 0.004	0.983 ± 0.003

**Fig. 4:**
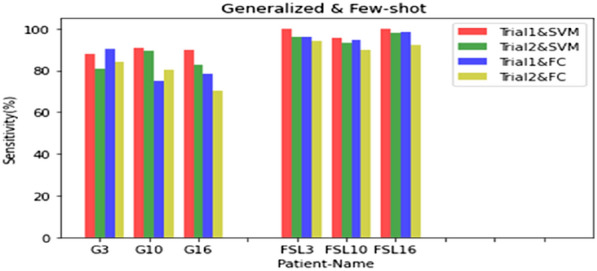
Sensitivity chart for three CHB–MIT patients with different methods and trials. (G3 = Generalizable method for patient Chb03, FSL3 = FSL method for patient Chb03)

To compare better the results of both methods, three patients who were not used in the generalizable method were tested with both methods and the results are shown in fig. 4. The sensitivity of the FSL method is better, especially when the SVM is used. For Shorter SPH, there is more sensitivity and there is a tradeoff between the SPH and the sensitivity. Averaged sensitivity for three patients in the 5-min prediction horizon and the 25-min seizure occurrence period is 98.52% and a false prediction rate of 0.045/h. These results easily show the outperformance of the proposed method in predicting seizures with only a few records.

## Discussion

The works done to predict epilepsy are either patient-specific or generalizable. The patient's specific methods are more accurate and the reason is to choose the best combination of features for that patient. Among the generalizable technique, those that have used deep neural networks, especially CNN, have more acceptable results. Most importantly, they do not require hand-crafted feature extraction and feature engineering. They also have better generalizability. However, all of the above methods require a lot of data. Recording the preictal EEG signal from an epileptic patient is complexed and annoying for the patient and in some cases impossible. In this work, we have presented a method called few-shot learning that is suitable for patients who have difficulty recording the preictal signal or may eventually have several signals available.

In this article, we first trained the network with data from 15 patients, and once we used SVM and once FC as a classifier, we froze the weights of CNN layers. In the next step, we tuned the above network for the data of a new patient, and in fact, we updated the weights of the classifier layers.

The proposed FSL method, having the prior knowledge gained from the generalizable method, is adjusted for the patient with only a few samples of preictal EEG signal from the new patient. This method reduces the effort to collect more data. Figure 4 shows that the accuracy obtained in this method is higher than generalizable methods. In addition to the fact that there is no need for long recordings, another advantage of this method over the patient-specific method is that it does not require hand crafted feature extraction, feature selection and model personalization, and it can be changed quickly for a new patient.

In this work the evaluation results showed a mean sensitivity of 98.52% and FPR = 0.045/h for the 5 min prediction horizon and the 25 min seizure occurrence period which is improved compared to previous works [7] with an equal forecast horizon. For the 10 min SPH and the 20 min SOP, we reached an average sensitivity of 95.70% and FPR = 0.057/h. In the references [33–35], the preictal interval is considered exactly at the beginning of the ictal interval, which means SPH = 0, while the higher the forecast horizon, the better. Tables 1, 2, 3, and 4 show that the higher the SPH, the lower the sensitivity. In the proposed method, the SPH is relatively high, about 5–10 min, which is suitable for the patient's preventive measures, and the SOP is low, about 20–25 min, which makes the patient wait less for occurrence and have less anxiety. In Table 5, we see that tasks [16, 20, 32, 36, 37] are less sensitive than our work and also have a higher false prediction rate per hour, which shows the superiority of our FSL method. Examining the results in both methods shows that SVM instead of FC is better for classification. The use of the SVM classifier, in trial 1 and trial 2 has increased the sensitivity by 2.71% and 3.50%. Figure 4 shows that the test results of the FSL method with three seizures from the Chb03 patient are more accurate than the test results of the generalizable method on the same patient. The results have also improved for the Chb10 and Chb16 patients. By comparing the results, we showed that setting up a generalizable method for a specific patient by the FSL method is more efficient and accurate. To compare the obtained results with some state-of-the-art, in Table 5, which show that there is relatively good sensitivity and FPR for this prediction horizon in our work.

**Table 5:** Comparison of the results of the state-of-the-art

Authors	Method	Database	Sensitivity (%)	FPR(/h)	SOP(min)	SPH(sec)
2017 [32]	Phase locking value + SVM	23Chb	82.44	–	5	0
2018 [16]	Zero crossings, PSD + LSTM	23Chb	90	0.11–0.02	15–120	0
2018 [7]	STFT + CNN	13Chb	81.4	0.16	30	300
2017[33]	CSP + LDA	24Chb	89	0.39	120	0
2019 [34]	spectral Power + 3DCNN	16Chb	85.7	0.096	60	0
2018 [20]	Wavelet transform + CNN	23Chb	87.8	0.14	10	0
2019 [35]	Raw EEG + Bi-LSTM	22Chb	99.72	0.004	60	0
2020 [36]	CNN + ELM	23Chb	95.85	0.045	–	–
2021 [37]	STFT + RDANet	13 Chb	89.33	–	–	–
**This work**	Raw EEG + CNN	15Chb	88.49	0.114	20–25	300–600
	**FEW-SHOT LEARNING**	**1Chb**	**98.52**	**0.045**	**20–25**	**300–600**

In this work the evaluation results showed a mean sensitivity of 98.52% and FPR=0.045/h for the 5 minute prediction horizon and the 25 minute seizure occurrence period which is improved compared to previous works [7] with an equal forecast horizon

The ideal work in the field of epilepsy prediction is to reach high sensitivity and low FPR on a high seizure prediction horizon (SPH) and a low seizure occurrence period (SOP). Our main goal in this work is epileptic patients in whom seizures occur late and there is not much data available about them. The above proposed method is trained with only a small number of samples and the results are closer to the ideal compared to other works.

## Conclusions

Seizure prediction allows the patient to take effective and preventive measures and also make a variety of treatments for patient possible. For example, instead of continuous medication that causes neurological complications, treatment can only be given at the necessary times when the onset is likely to occur. For example, patients who are taking persistent antiepileptic drugs can take seizure drugs, such as episodic ones. We used EEG signals that do not require surgery and are recorded on the scalp for this task. In this paper, a new few-shot learning perspective was proposed to predict epileptic seizures based on multi-channel raw EEG signals. In the proposed method, recording a long signal is not required. The method works with low amount of data, short time, without employing an expert, is adjustable for each new patient and is more efficient compared to patient-specific methods and other generalizable methods that were examined. This study provided a promising solution for seizure prediction with multi-channel raw EEG for patients with a low frequency of seizures or even for patients who do not have the conditions for long-term recording of EEG signals. In the next works, we will use different data sets in different age ranges along with data set CHB–MIT, which is mostly related to children, to further evaluate the efficiency of our model.

## Data Availability

https://physionet.org/content/chbmit/1.0.0/chb20/.
